# Vitamin D Deficiency and Cardiovascular Disease Risk Factors Among American Indian Adolescents: The Strong Heart Family Study

**DOI:** 10.5888/pcd22.240354

**Published:** 2025-04-04

**Authors:** Jessica A. Reese, Erin Davis, Amanda M. Fretts, Tauqeer Ali, Elisa T. Lee, Jason G. Umans, Ronit Yarden, Ying Zhang, Jennifer D. Peck

**Affiliations:** 1 Center for American Indian Health Research, Hudson College of Public Health, University of Oklahoma Health Sciences Center, Oklahoma City; 2 Department of Biostatistics and Epidemiology, Hudson College of Public Health, University of Oklahoma Health Sciences Center, Oklahoma City; 3 Department of Anatomical Sciences and Neurobiology, University of Louisville, Louisville, Kentucky; 4 Department of Epidemiology, University of Washington School of Public Health, Seattle; 5 MedStar Health Research Institute, Hyattsville, Maryland; 6 Georgetown-Howard Universities Center for Clinical and Translational Science, Washington, DC; 7 Division of Cardiovascular Sciences, National Heart, Lung, and Blood Institute, Bethesda, Maryland

## Abstract

**Introduction:**

We aimed to describe the prevalence of vitamin D deficiency among American Indian adolescents and determine its association with cardiovascular disease (CVD) risk factors.

**Methods:**

Our study population consisted of 307 adolescents (aged ≤20 years) participating in the Strong Heart Family Study with serum 25-hydroxyvitamin D (25[OH]D) measured on samples collected during baseline examinations (2001–2003). We defined baseline prevalence of vitamin D deficiency as 25(OH)D ≤20 ng/mL. We evaluated outcomes related to obesity (BMI, waist circumference, wait-to-hip ratio, and body fat percentage), diabetes, cholesterol, and metabolic syndrome. We used generalized estimating equations to determine whether the prevalence of the outcomes differed according to vitamin D deficiency status, while controlling for covariates. To determine incidence, we conducted a follow-up examination a median 5.8 years after baseline (2006–2009) and a second follow-up a median of 13.3 years after baseline (2014–2018). We calculated incidence rates (IR) per 100 person-years for the total group and stratified by vitamin D deficiency status at baseline. Finally we used shared frailty cox proportional hazards models to determine if the risk of the outcomes differed according to vitamin D deficiency status, while controlling for covariates.

**Results:**

The prevalence of vitamin D deficiency was 50.8% at baseline, and it was associated with the prevalence of obesity, low HDL-C, and metabolic syndrome, while controlling for covariates. By the first follow-up, the IRs per 100 person-years were the following: obesity (5.03), diabetes (1.07), any dyslipidemia (10.80), and metabolic syndrome (3.31). By the second follow-up, the IR of diabetes was significantly higher among those with (vs without) baseline vitamin D deficiency (1.32 vs 0.68 per 100 person-years; *P* = .02), although the association was not significant after adjusting for covariates.

**Conclusion:**

Vitamin D deficiency in adolescence may be associated with the CVD risk factors obesity, low HDL-C, and metabolic syndrome and may also contribute to the development of diabetes later in life.

**Summary**

**What is already known on this topic?**

In non-Native populations in the US, vitamin D deficiency is associated with obesity prevalence and may be amenable to interventions through changes in diet and vitamin supplementation.

**What is added by this report?**

This first report of vitamin D deficiency prevalence among American Indian adolescents and its association with cardiovascular disease risk factors demonstrated an independent association between the prevalence of metabolic syndrome and vitamin D deficiency. Thirteen years after baseline, the incidence rate of diabetes was significantly higher among American Indian adolescents with (vs without) vitamin D deficiency.

**What are the implications for public health practice?**

These results may provide a path for developing measures to reduce cardiovascular disease risk factors at an early age in American Indians.

## Introduction

According to National Health and Nutrition Examination Survey (NHANES) data for 2001 through 2018, only 25.5% to 27.5% of US adolescents have sufficient serum 25-hydroxyvitamin D (25[OH]D) (1). Racial and ethnic differences in 25(OH)D levels exist, with the prevalence of deficiency higher among non-Hispanic Black and Hispanic groups than non-Hispanic White groups (1,2). However, information on 25(OH)D levels among American Indian adolescents is limited to a single study, which reported a mean (SD) 25(OH)D level of 17.8 (0.4) ng/mL (deficient) in a population of American Indian children and adolescents (aged 5–18 y) in Nebraska (3).

Vitamin D deficiency may be associated with obesity and other cardiovascular disease (CVD) risk factors, such as dyslipidemia and diabetes among adults (4,5). Associations have been observed between vitamin D deficiency and obesity, elevated hypertension, low high-density lipoprotein cholesterol (HDL-C), and diabetes among children, adolescents, and young adults (aged 1–21 y) (6). However, these associations are largely from cross-sectional studies; the temporal relationship between vitamin D deficiency and CVD risk factors remains to be determined. Additionally, no current studies have addressed associations between vitamin D deficiency and CVD risk factors in the American Indian adolescent population (7).

This study aimed to address this gap by using Strong Heart Family Study (SHFS) data to establish the baseline prevalence of vitamin D deficiency among American Indian adolescents. Because low levels of 25(OH)D are amenable to interventions through diet, vitamin supplementation, and lifestyle modifications, if temporal relationships between vitamin D deficiency and CVD risk factors exist, there is potential to reduce and control obesity and other CVD-related factors at a young age (8). Therefore, the objectives of this study were to evaluate the cross-sectional associations between vitamin D deficiency and CVD risk factors, as well as associations between vitamin D deficiency and incident obesity, diabetes, dyslipidemia, and metabolic syndrome, among American Indian adolescents who participated in SHFS.

## Methods

All data were collected, analyzed, and reported under agreements made with the sovereign tribal nations that partnered in this research; the agreements preclude commonly accepted modes of data sharing. Requests to access the data set from qualified researchers trained in human subject confidentiality protocols may be sent to the Strong Heart Study Coordinating Center at https://strongheartstudy.org. Requests will be reviewed by tribal research partners before data can be released. This policy is consistent with the NIH Policy for Data Management and Sharing: Responsible Management and Sharing of American Indian/Alaska Native Participant Data (9).

### Study population

SHFS is a multicentered, family-based, prospective cohort study of CVD among American Indians (10,11). It includes 12 American Indian communities and tribes living in central Arizona, southwestern Oklahoma, and North and South Dakota (10). Participants include the original Strong Heart Study cohort members, their extended family members, and additional families from the same regions and communities. For this analysis, we included adolescents who participated in the baseline examination (2001–2003) of the SHFS (11,12). We used information collected at baseline to determine prevalence measures of obesity, diabetes, any dyslipidemia, metabolic syndrome, and covariates (13,14). We invited all baseline participants to participate in a follow-up examination (2006–2009; median [range] years after baseline = 5.8 [3.0–8.5]) (11). We also performed a second follow-up (2014–2018; median [range] years after baseline = 13.3 [11.1–15.5]). This second follow-up was limited in that it included only collection of survey data (demographic and medical history questionnaire) and a medical record review for selected variables, including diabetes. We did not assess obesity, dyslipidemia, or metabolic syndrome at the second follow-up because we did not perform a physical examination. Information collected at baseline and follow-up was used to determine the incidence of obesity, diabetes, any dyslipidemia, and metabolic syndrome (13,14).

### 25(OH)D assessment

At the time of baseline recruitment, we collected and stored blood samples in a −80° C freezer. During an SHFS ancillary study in 2014, we used tandem mass spectrometry to measure the predominant circulating form of vitamin D, 25(OH)D, on blood collected during the baseline examination; this measurement took place 11 to 13 years after baseline data collection. We defined vitamin D deficiency according to the Institute of Medicine–recommended serum cut points for 25(OH)D: deficient is defined as ≤20 ng/mL (≤50 nmol/L) and sufficient as >20 ng/mL (>50 nmol/L) (15).

### Obesity assessment

At both baseline and follow-up, we assessed height, weight, waist, and hip circumference during the physical examination. We measured weight with a Tanita BWB-800 5 adult digital scale and height with a vertical mounted ruler (16). We calculated body mass index (BMI) by dividing weight in kilograms by height in meters squared (kg/m^2^) (17). At baseline, because all participants were adolescents, we defined obesity as the 95th percentile and overweight as the 85th percentile of BMI based on age, definitions developed by the National Center for Health Statistics (Table 1) (18). At follow-up, when all participants were adults, we defined obesity as BMI ≥30 kg/m^2^ and overweight as BMI equal to 25.0–29.9 kg/m^2^ (17).

**Table 1:** Definitions of Baseline and Follow-Up Cardiovascular Disease Risk Factors Used in the Strong Heart Family Study^a^

Risk factor	Baseline definition	Follow-up definition
**Overweight and obesity**		
Obese	BMI ≥95th percentile based on age	BMI ≥30kg/m^2^
Overweight or obese	BMI ≥85th percentile based on age	BMI ≥25 kg/m^2^
**High waist circumference**		
Male	≥39.2 in for age 15 y	≥40 in
	≥39.6 in for age 16 y	
	≥39.9 in for age 17 y	
	≥40.0 in for age 18 y	
	≥40.0 in for age 19 y	
Female	≥33.1 in for age 15 y	≥35 in
	≥33.5 in for age 16 y	
	≥33.9 in for age 17 y	
	≥34.3 in for age 18 y	
	≥34.5 in for age19 y	
**High waist-to-hip ratio**		
Male	≥0.90	≥0.90
Female	≥0.85	≥0.85
**High body fat percentage**		
Male	≥25%	≥25%
Female	≥35%	≥35%
**Impaired FPG**	≥110 to <126 mg/dL	≥110 to <126 mg/dL
**Diabetes**	FPG ≥126 mg/dL and/or taking diabetes medication	FPG ≥126 mg/dL and/or taking diabetes medication
**High total cholesterol**	≥200 mg/dL	≥200 mg/dL
**High LDL-C**	≥100 mg/dL	≥100 mg/dL
**Low HDL-C**		
Male	≤40.2 mg/dL for age 15 y	≤40 mg/dL
	≤39.8 mg/dL for age 16–20 y	
Female	≤48.7 mg/dL for age 15 y	≤50 mg/dL
	≤49.1 mg/dL for age 16–17 y	
	≤49.5 mg/dL for age 18 y	
	≤49.9 mg/dL for age19 y	
**High non-HDL-C**	≥130 mg/dL	≥130 mg/dL
**High triglycerides**		
Male	≥138 mg/dL for age 15 y	≥150 mg/dL
	≥141 mg/dL for age 16 y	
	≥143 mg/dL for age 17 y	
	≥146 mg/dL for age 18 y	
	≥149 mg/dL for age 19 y	
Female	≥127 mg/dL for age 15 y	≥150 mg/dL
	≥129 mg/dL for age 16 y	
	≥135 mg/dL for age 17 y	
	≥142 mg/dL for age 18 y	
	≥149 mg/dL for age19 y	
**Any dyslipidemia**	Any abnormal value of total cholesterol, LDL-C, HDL-C, non-HDL-C, or triglycerides, listed above, and/or taking lipid medication	Any abnormal value of total cholesterol, LDL-C, HDL-C, non-HDL-C, or triglycerides, listed above, and/or taking lipid medication
**High blood pressure, mm Hg**		
Male	>126/81 for age 15 y	>140/90 mm Hg
	>128/82 for age 16 y	
	>128/83 for age 17 y	
	>129/84 for age 18 y	
	>130/85 for age 19 y	
Female	>126/84 for age 15 y	
	>128/84 for age 16 y	
	>128/85 for age 17 y	
	>129/85 for age 18 y	
	>130/85 for age 19 y	
**Hypertension**	High blood pressure based on the above criteria, and/or taking antihypertensive medication	High blood pressure based on the above criteria, and/or taking antihypertensive medication
**Albuminuria**	Albumin-creatinine ratio ≥30 mg/g	Albumin-creatinine ratio ≥30 mg/g
**Metabolic syndrome**	Any 3 of the following: high waist circumference, high blood pressure, high triglycerides, or low HDL-C, based on the criteria above, or FPG ≥100 mg/dL	Any 3 of the following: high waist circumference, high blood pressure, high triglycerides, or low HDL-C, based on the criteria above, or FPG ≥100 mg/dL

Abbreviations: BMI, body mass index; FPG, fasting plasma glucose; HDL-C, high-density lipoprotein cholesterol; LDL-C, low-density lipoprotein cholesterol.

a At baseline, because all participants were adolescents, we used age- and sex-specific cutoffs when they existed; otherwise, we used adult cutoffs. Because all participants were adults at follow-up, we used adult cutoffs.

Similarly, at baseline we defined high waist circumference on the basis of age- and sex-specific cutoffs for adolescents (19) and at follow-up as >40 in for men or >35 in for women (20). We calculated waist-to-hip ratio by dividing the waist circumference by the hip circumference. We defined high waist-to-hip ratio as ≥0.90 for males and ≥0.85 for females at baseline and follow-up. We used an impedance meter (model B14101, RJL Equipment Co) to estimate body mass and used equations based on total body water validated in American Indian populations (21). We defined high body fat as ≥25% for males and ≥35% for females at baseline and follow-up (21). We defined incident high BMI, high waist circumference, high waist-to-hip ratio, or high body fat as the development by the first follow-up examination among participants who did not have these conditions at baseline.

### Diabetes assessment

We defined diabetes as taking diabetes medication, and/or having a fasting plasma glucose (FPG) level ≥126 mg/dL (22). We defined impaired fasting glucose (IFG) as an FPG from 110 mg/dL to <126 mg/dL (16,22). To measure FPG, we drew blood after a 12-hour fast at baseline and first follow-up (23). In addition, to determine the use of medications for diabetes at baseline and the first follow-up, we asked participants to bring their medications to the physical examination and to recall (with assistance from an adult for minors) additional medications (24). At second follow-up, based on medical record review, we classified a participant as having diabetes if FPG ≥126 mg/dL, hemoglobin A_1c_ ≥6.5%, 2-hour plasma glucose during an oral glucose tolerance test ≥200 mg/dL, or the participant was using insulin, oral agents, or diet and/or exercise for diabetes treatment. We defined incident diabetes as the development of diabetes by the first or second follow-up among participants who did not have diabetes at baseline.

### Dyslipidemia assessment

To measure lipids, we drew blood after a 12-hour fast at baseline and first follow-up examination (14,23). At baseline, abnormal cholesterol was based on age- and sex-specific cutoffs for adolescents (19) and at follow-up based on sex-specific cutoffs for adults (Table 1). We defined any dyslipidemia as high total cholesterol, high low-density lipoprotein cholesterol (LDL-C), low HDL-C, high non-HDL-C, high triglycerides, or taking lipid-lowering medication (Table 1) (14,25). We defined dyslipidemia incidence as the development of any dyslipidemia by the first follow-up examination among participants who did not have dyslipidemia at baseline.

### Metabolic syndrome assessment

We defined metabolic syndrome as having at least 3 of the 5 components for the syndrome: high waist circumference, high blood pressure, high triglycerides, high FPG, or low HDL-C. At baseline, we used age- and sex-specific cutoffs, and at follow-up, we used adult cutoffs (Table 1) (13,14). We measured blood pressure in the right arm while the participant was in the seated position after 5 minutes of rest, and we used the average of the second and third measurements for analysis (16,24). We defined metabolic syndrome incidence as the development of metabolic syndrome by the first follow-up examination among participants who did not have it at baseline.

### Covariate assessment

We selected several covariates, which investigations previously reported to be associated with both vitamin D deficiency and the CVD risk factor outcomes (7,24,26). During the baseline and follow-up examinations, we collected self-reported data on demographic and clinical characteristics (age, sex, and current smoking) (14). At both baseline and first follow-up, we defined hypertension as having high blood pressure (Table 1) and/or taking antihypertension medication. We estimated renal function by using the urinary albumin-creatinine ratio and defined albuminuria as ≥30 mg/g.

To determine the amount of vitamin D intake at baseline, we administered a Block 119-item food frequency questionnaire (FFQ) (27). In addition to the questions on the standard Block FFQ, we included supplemental questions about consumption of common American Indian foods, such as menudo, pozole, guava, red or green chili, Indian taco, fry bread, corn tortilla, flour tortilla, and Spam (16,28). For each standard and supplemental food item listed on the FFQ, participants reported how often they consumed each in the previous year, consumption frequency (never, a few times per year, once per month, 2 or 3 times per month, once per week, twice per week, 2 or 3 times per week, 5 or 6 times per week, or daily), and portion size (small, medium, or large) (7,16). We used the Block database (Block Dietary Systems) to calculate micronutrient intakes, including vitamin D (28).

### Statistical analysis

We used descriptive statistics to summarize the prevalence of 25(OH)D according to the standardized cut point (20 ng/mL). For the baseline cross-sectional analysis, we reported the mean (SD) for normally distributed continuous variables, the median (IQR) for skewed variables, or the frequency and percentage for categorical variables. Because of the familial sampling design, our data were correlated. Therefore, we used generalized estimating equation (GEE) methods to determine whether risk factors at baseline differed between participants with and without vitamin D deficiency, while accounting for clustering between families. Since vitamin D deficiency may vary according to sunlight exposure, we used study center as a surrogate for sunlight exposure. We summarized the prevalence of vitamin D deficiency at each study location and for all 3 centers combined. In addition, we evaluated the season of serum collection as a surrogate for sunlight exposure.

To evaluate the cross-sectional association while controlling for covariates and accounting for the clustered family sampling study design, we used GEE methods to estimate multivariable logistic regression models and calculate prevalence odds ratios (PORs) and 95% CIs. We selected covariates (age, sex, study center, current smoking, hypertension, BMI percentile, diabetes, or any dyslipidemia) on the basis of previously reported associations with vitamin D deficiency and outcomes (7,24,26). All selected covariates were simultaneously entered in the multivariable models. Because metabolic syndrome is a combined outcome containing measures of obesity, lipids, blood pressure, and FPG, we adjusted the metabolic syndrome model for age, sex, study center, and current smoking.

To explore how baseline 25(OH)D levels may influence future CVD risk factors, we analyzed the incidence of CVD risk factors. We defined the incidence of CVD risk factors as the development of the risk factor, based on age- and sex-specific cut points, among participants who did not have the risk factor at baseline. After we made baseline exclusions for each outcome, we calculated the incidence rate (IR) per 100 person-years for the total group and stratified by vitamin D deficiency status at baseline. We used Kaplan–Meier curves and log-rank tests to determine whether the probabilities of each CVD risk factor outcome differed between participants with or without baseline vitamin D deficiency (29).

We assessed the multivariable relationship between vitamin D deficiency and incident CVD risk factors by using a shared frailty Cox model based on proportional hazards to account for the relatedness among participants (29). We used this method to calculate hazard ratios (HRs) and 95% CIs of associations between vitamin D deficiency and time to each incident CVD risk factor. Each reported analysis met the assumption of proportional hazards. We used similar model-building procedures as we used in the cross-sectional analyses. Interaction between covariates and any dyslipidemia was evaluated by including appropriate cross-product terms in the model, and no significant interactions were found. We also considered models with season of blood draw as a covariate (spring/summer vs fall/winter), waist circumference instead of BMI as a covariate (in the diabetes and dyslipidemia models), and continuous outcomes instead of categorical outcomes. The outcomes of these analyses were not meaningfully different than those presented. We used a significance level of .05 for hypothesis tests and performed statistical analyses in SAS version 9.4 (SAS Institute Inc).

## Results

### Population characteristics and prevalence of vitamin D deficiency

At baseline, 320 participants met the inclusion criteria for being aged 20 years or younger. Of these, 307 (95.9%) had valid 25(OH)D measurements. Of these 307, 38 (12.4%) did not participate in the first follow-up examination. The mean (SD) age at baseline was 17.4 (1.5) years; 52.1% were female, 25.5% were smokers, 10.0% had hypertension, and none had albuminuria (Table 2). Median (IQR) vitamin D intake was 127.8 (61.9–267.3) IU, and 9.8% were taking vitamin D supplements. Of the 307 participants at baseline, 156 (50.8%) had vitamin D deficiency. When stratified by study center, the prevalence of vitamin D deficiency was 80.0% in Arizona, 40.6% in Oklahoma, and 48.4% in the Dakotas (*P* < .001). The prevalence of vitamin D deficiency was significantly higher when data were collected in fall or winter than when collected in spring or summer (59.5% [50 of 84] vs 47.5% [106 of 223]; *P* = .03).

**Table 2:** Comparison of Baseline Demographic and Cardiovascular Disease Risk Factors Among American Indian Adolescents, Stratified by Vitamin D Deficiency Status,^a^ Strong Heart Family Study, 2001–2003^b^

Variable at baseline	Total (N = 307)	Vitamin D deficiency status		
		Deficient (n = 156)	Not deficient (n = 151)	*P* value^c^
**Age, mean (SD), y**	17.4 (1.5)	17.6 (1.5)	17.2 (1.4)	.06
**Sex, no. (%)**				
Female	160/307 (52.1)	99 (61.9)	61 (38.1)	<.001
Male	147/307 (47.9)	57 (38.8)	90 (61.2)	
**Center, no. (%)**				
Arizona	49/307 (16.0)	39 (80.0)	10 (20.0)	<.001
Oklahoma	101/307 (32.9)	41 (40.6)	60 (59.4)	
North and South Dakota	157/307 (51.1)	76 (48.4)	81 (51.6)	
**Smokes, no. (%)**	78/306 (25.5)	48 (61.5)	30 (38.5)	.01
**Hypertension**				
Has hypertension, no. (%)^d^	31/307 (10.1)	18 (58.1)	13 (41.9)	.34
Takes hypertension medication, no. (%)	1/307 (0.3)	0	1 (100.0)	—e
**Blood pressure, mean (SD), mm Hg**				
Systolic	113.0 (10.9)	113.8 (10.5)	112.8 (11.2)	.79
Diastolic	69.0 (9.9)	70.9 (9.3)	67.8 (10.3)	.03
**Albuminuria, no. (%) **	0	0	0	—e
**Plasma creatinine, mean (SD), mg/dL**	0.8 (0.1)	0.7 (0.1)	0.8 (0.1)	<.001
**Vitamin D**				
Intake, median (IQR), IU	127.8 (61.9–267.3)	110.3 (52.8–207.8)	142.2 (84.1–349.9)	.007
Takes vitamin D supplements, no. (%)	29/296 (9.8)	14 (48.3)	15 (51.7)	.60
**Data collected in fall or winter, no. (%)**	84/307 (27.4)	50 (59.5)	34 (40.5)	.03
**Obesity**				
**Overweight and obesity^d^**				
BMI ≥ 85th percentile (overweight or obese)	161/306 (52.6)	99 (61.5)	62 (38.5)	.001
BMI ≥ 95th percentile (obese)	103/306 (33.7)	70 (68.0)	33 (32.0)	<.001
**Waist circumference**				
Measurement, mean (SD), inches	36.0 (7.1)	37.9 (7.2)	34.0 (6.3)	<.001
Has high waist circumference, no. (%)^d^	136/305 (44.6)	93 (68.4)	43 (31.6)	<.001
**Waist-to-hip ratio**				
Ratio, mean (SD)	0.9 (0.1)	0.9 (0.1)	0.8 (0.1)	.005
Has high ratio, no. (%)^d^	128/304 (42.1)	84 (65.6)	44 (34.4)	<.001
**Body fat**				
Body fat percentage, mean (SD)	31.9 (11.6)	36.3 (11.6)	27.3 (9.7)	<.001
Has high body fat percentage, no. (%)^d^	162/303 (53.4)	108 (66.7)	54 (33.3)	<.001
**Diabetes**				
Fasting plasma glucose, mean (SD), mg/dL	91.7 (17.1)	91.7 (15.9)	91.6 (18.2)	.97
Has diabetes, no. (%)	6/307 (2.0)	4 (66.7)	2 (33.3)	.38
Has diabetes or impaired fasting glucose, no. (%)	42/307 (13.7)	21 (50.0)	21 (50.0)	.78
Takes diabetes medication, no. (%)	3/307 (1.0)	2 (66.7)	1 (33.3)	.38
**Lipids**				
Total cholesterol, mean (SD), mg/dL	154.4 (30.0)	153.8 (30.6)	155.1 (29.4)	.34
Has high total cholesterol, no. (%)	25/307 (8.1)	16 (64.0)	9 (36.0)	.34
LDL-C, mean (SD), mg/dL	82.9 (24.6)	82.6 (25.6)	83.1 (23.7)	.50
Has high LDL-C, no. (%)	70/307 (22.8)	38 (54.3)	32 (45.7)	.86
HDL-C, mean (SD), mg/dL	49.4 (13.0)	47.9 (12.5)	51.0 (13.4)	.11
Has low HDL-C, no. (%)^d^	124/307 (40.4)	82 (66.1)	42 (33.9)	<.001
Non-HDL-C, no. (%), mg/dL	105.0 (30.9)	105.8 (31.7)	104.1 (30.2)	.80
Has high non-HDL-C, no. (%)	60/307 (19.5)	32 (53.3)	28 (46.7)	.66
Triglycerides, median (IQR), mg/dL	93.0 (73.0–133.0)	97.0 (77.0–141.5)	89.0 (70.0–119.0)	.26
Has high triglycerides, no. (%)^d^	66/307 (21.5)	42 (63.6)	24 (36.4)	.04
Takes lipid-lowering medication, no. (%)	0/307	0	0	—e
Has any dyslipidemia, no. (%)	172/307 (56.0)	104 (60.5)	68 (39.5)	<.001
**Composite cardiovascular disease risk factor**				
Has metabolic syndrome	52/305 (17.0)	34 (65.4)	18 (34.6)	.02

Abbreviations: 25(OH)D, serum 25-hydroxyvitamin D; BMI, body mass index; HDL-C, high density lipoprotein cholesterol; LDL-C, low-density lipoprotein cholesterol.

a Vitamin D deficiency defined as 25(OH)D ≤20 ng/mL.

b Baseline measurements were taken during 2001–2003.

c Determined from generalized estimating equations, controlling for familial clustering; *P* <.05 considered significant.

d Based on age-specific and sex-specific cut points for adolescents.

e Sample size not adequate to generate a *P* value.

### Cross-sectional associations between vitamin D deficiency and CVD risk factors

In the cross-sectional analysis at baseline of associations between vitamin D deficiency and measures of obesity, the frequencies of all outcome measures of obesity were higher among participants with vitamin D deficiency than among participants with sufficient 25(OH)D levels (all *P* values < .01, Table 2). These outcome measures included the prevalence of obesity, overweight or obesity percentile, high waist circumference, high waist-to-hip ratio, and body fat percentage. We found no significant difference in diabetes measures between participants with (vs without) vitamin D deficiency. Finally, the prevalence of the following outcome measures was significantly higher among participants with vitamin D deficiency than among participants without the deficiency: low HDL-C (66.1% vs 33.9%, *P* < .001), high triglycerides (63.6% vs 36.4%, *P* = .04), any dyslipidemia (60.5% vs 39.5%, *P* < .001), and metabolic syndrome (65.4% vs 34.6%, *P* = .02).

**Figure 1:**
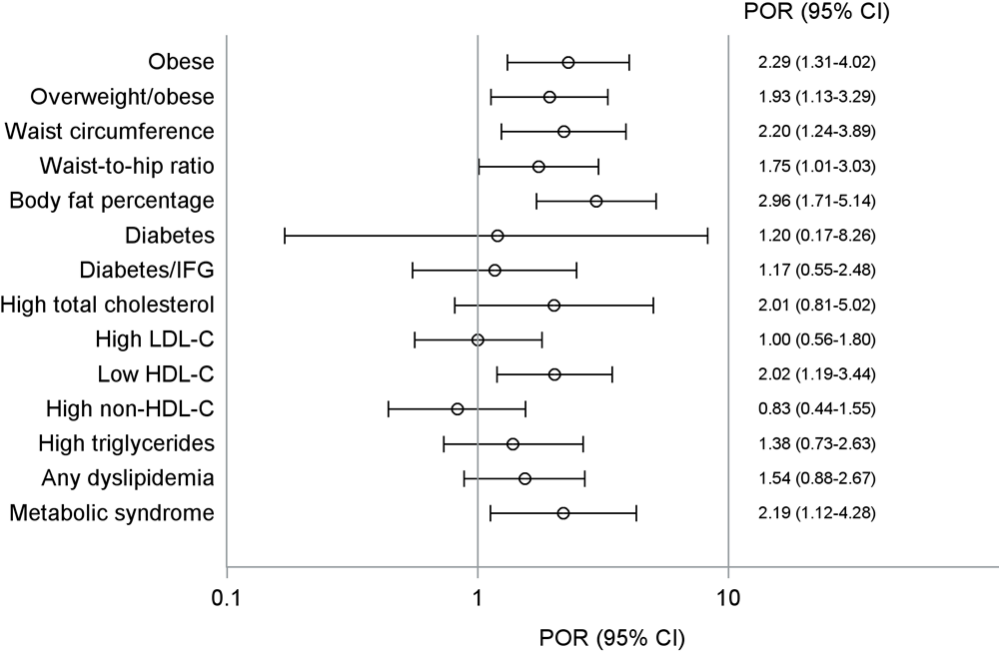
Baseline cross-sectional association between prevalence of vitamin D deficiency and prevalence of CVD risk factors among American Indian adolescents, Strong Heart Family Study. All models accounted for the correlated family structure; see text for definitions of risk factors and details on how models were adjusted. Abbreviations: HDL-C, high-density lipoprotein cholesterol; IFG, impaired fasting glucose; LDL-C, low-density lipoprotein cholesterol; POR, prevalence odds ratio.

In the assessment of the multivariable relationship between vitamin D deficiency and prevalence of CVD risk factors, controlling for age, sex, study center, smoking, hypertension, diabetes, and any dyslipidemia, the odds of all measures of obesity were higher among American Indian adolescents with vitamin D deficiency than among those without the deficiency (*P* value for all outcomes < .05). The odds of prevalent low HDL-C were twice as high (POR = 2.02, 95% CI, 1.19–3.44), controlling for age, sex, study center, current smoking, hypertension, BMI ≥95th percentile, and diabetes (Figure 1). Finally, the odds of prevalent metabolic syndrome were a little over twice as high (POR = 2.19, 95% CI, 1.12–4.28), controlling for age, sex, study center, and smoking (Figure 1).

**Table:** 

Risk factor	POR (95% CI)
Obese	2.29 (1.31–4.02)
Overweight or obese	1.93 (1.13–3.29)
Waist circumference	2.20 (1.24–3.89)
Waist-to-hip ratio	1.75 (1.01–3.03)
Body fat percentage	2.96 (1.71–5.14)
Diabetes	1.20 (0.17–8.26)
Diabetes or impaired fasting glucose	1.17 (0.55–2.48)
High total cholesterol	2.01 (0.81–5.02)
High LDL-C	1.00 (0.56–1.80)
Low HDL-C	2.02 (1.19–3.44)
High non-HDL-C	0.83 (0.44–1.55)
High triglycerides	1.38 (0.73–2.63)
Any dyslipidemia	1.54 (0.88–2.67)
Metabolic syndrome	2.19 (1.12–4.28)

### Associations between vitamin D deficiency and incidence of CVD risk factors

**Figure 2:**
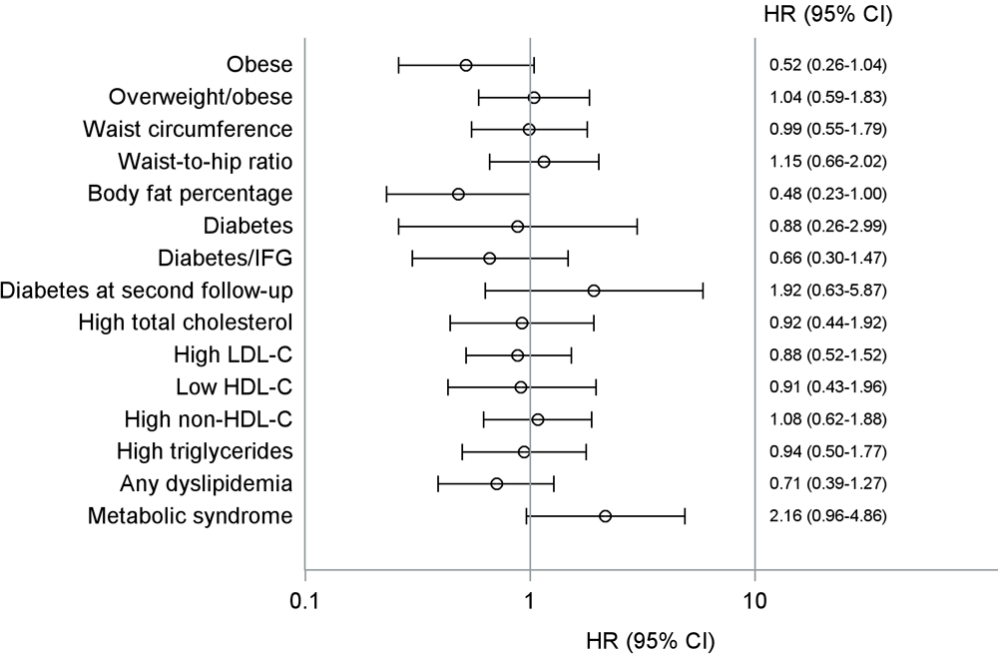
Association between vitamin D deficiency and development of cardiovascular disease risk factors among American Indian adolescents, Strong Heart Family Study. For each model, those who had the risk factor at baseline were excluded, and all outcomes were directly measured at the first follow-up (except for diabetes) at second follow-up. All models accounted for the correlated family structure; see text for definitions of risk factors and details on how models were adjusted. Abbreviations: HR, hazard ratio; HDL-C, high-density lipoprotein cholesterol; IFG, impaired fasting glucose; LDL-C, low-density lipoprotein cholesterol.

Because we made exclusions at baseline for each outcome and the prevalence of each factor at baseline differed, the sample size differed for each outcome (Table 3). At first follow-up, sample sizes ranged from 115 participants for any dyslipidemia to 257 participants for diabetes. Likewise, the IR per 100 person-years of each outcome ranged from 1.02 for diabetes at second follow-up (median 13.3 y after baseline) to 10.80 for any dyslipidemia at first follow-up (median 5.8 y after baseline). When stratifying by vitamin D deficiency status at baseline, the IRs for CVD risk factors were higher (but not significant) for participants with vitamin D deficiency at baseline, except for obesity, high body fat percentage, and any dyslipidemia, where the IR was slightly lower for participant vitamin D deficiency (but also not significant). Finally, the univariate IR per 100 person-years of diabetes at second follow-up was significantly higher among American Indian adolescents with vitamin D deficiency at baseline compared with those without vitamin D deficiency (1.32 vs 0.68; *P* = .02) (Table 3). In the multivariable analysis controlling for covariates, the risk of developing CVD risk factors was not significantly different among American Indian adolescents stratified by vitamin D deficiency status (Figure 2).

**Table 3:** Incidence of Cardiovascular Disease Risk Factors at First Follow-Up Among American Indian Adolescents, Stratified by Baseline Vitamin D Deficiency Status,^a^ Strong Heart Family Study^b^

Risk factor	No. of participants without risk factor at baseline^c^	Incidence rate per 100 person-years			*P* value^d^
		Total	Deficient	Not deficient	
**Obesity**					
Obese (BMI ≥30 kg/m^2^)^e^	178	5.03	4.82	5.19	.46
Overweight or obese (BMI ≥25 kg/m^2^)^e^	126	9.14	10.09	8.53	.50
High waist circumference (>40 in for males, >35 in for females)^e^	146	6.63	7.45	6.16	.84
High waist-to-hip ratio (≥0.9 for males, ≥0.85 for females)	153	10.07	11.69	8.93	.52
High body fat percentage (≥25% males, ≥35% females)	123	9.10	8.11	9.63	.31
**Diabetes**					
Has diabetes	257	1.07	1.34	0.80	.34
Has diabetes or impaired fasting glucose	228	3.30	3.41	3.19	.91
Has diabetes at second follow-up	242^f^	1.02	1.32	0.68	.02
**Lipids**					
Has high total cholesterol (≥200 mg/dL)	243	2.97	3.02	2.91	.91
Has high LDL-C (≥100 mg/dL)	203	6.24	6.28	6.19	.68
Has low HDL-C (≤40 mg/dL for males, ≤50 for females)^e^	154	4.60	5.22	4.17	.57
Has high non-HDL-C (≥130 mg/dL)	212	6.03	6.35	5.72	.82
Has high triglycerides (≥150 mg/dL)^e^	205	4.63	5.03	4.28	.97
Any dyslipidemia	115	10.80	9.44	11.65	.27
**Composite cardiovascular disease risk factor**					
Metabolic syndrome	214	3.31	6.42	3.86	.13

Abbreviations: 25(OH)D, serum 25-hydroxyvitamin D; BMI, body mass index, LDL-C, low-density lipoprotein cholesterol, HDL-C, high-density lipoprotein cholesterol.

a Vitamin D deficiency defined as 25(OH)D ≤20 ng/mL.

b Baseline variables were measured during 2001–2003, the first follow-up variables were measured during 2006-2009, and the second follow-up variable was measured 2014-2018. All variables presented were measured during the first follow-up using direct measurements during physical examinations. Diabetes was the only variable measured during the second follow-up, which was assessed through medical record review.

c Includes participants without the risk factor at baseline who were not missing at follow-up.

d *P* values generated from univariate log-rank tests; *P* < .05 considered significant.

e Baseline measures for BMI, high waist circumference, low HDL-C, and high triglycerides are based on age-specific and sex-specific cut points for adolescents. Otherwise, categories are based on adult standards.

f Includes participants without the risk factor at baseline or first follow-up who were not missing at the second follow-up.

**Table:** 

Risk factor	HR (95% CI)
Obese	0.52 (0.26–1.04)
Overweight or obese	1.04 (0.59–1.83)
Waist circumference	0.99 (0.55–1.79)
Waist-to-hip ratio	1.15 (0.66–2.02)
Body fat percentage	0.48 (0.23–1.00)
Diabetes	0.88 (0.26–2.99)
Diabetes or IFG	0.66 (0.30–1.47)
Diabetes at second follow-up	1.92 (0.63–5.87)
High total cholesterol	0.92 (0.44–1.92)
High LDL-C	0.88 (0.52–1.52)
Low HDL-C	0.91 (0.43–1.96)
High non-HDL-C	1.08 (0.62–1.88)
High triglycerides	0.94 (0.50–1.77)
Any dyslipidemia	0.71 (0.39–1.27)
Metabolic syndrome	2.16 (0.96–4.86)

## Discussion

This is the first study to evaluate the potential association between vitamin D deficiency and the prevalence and incidence of CVD risk factors among American Indian adolescents. Half (50.8%) of American Indians that made up the study population had vitamin D deficiency in adolescence, which is more than twice that of non-Hispanic White adolescents from NHANES (1). Additionally, various indicators of obesity and adiposity, low HDL-C, and metabolic syndrome were more prevalent among participants with versus without vitamin D deficiency, although the prevalence of diabetes was similar between vitamin D deficiency groups at baseline. When adolescents without risk factors were followed prospectively for incident outcomes, we observed no evidence of associations between baseline vitamin D deficiency and subsequent development of obesity, dyslipidemia, diabetes, or metabolic syndrome after a median follow-up of 5.8 years. However, at 13-year follow-up for diabetes, the unadjusted IR was significantly higher for participants with baseline vitamin D deficiency versus without; however, the HR was not significant after adjusting for covariates. This finding may be due to a smaller sample size after making baseline exclusions and the relatively short follow-up time.

Inverse associations between 25(OH)D and CVD risk factors are well documented, although the causal mechanisms underlying these associations are not fully elucidated (30). Experimental evidence indicates that low levels of 25(OH)D may play a role in regulating gene expression or altering leptin and parathyroid hormones to influence obesity via adipose tissue differentiation and growth (30). Alternative hypotheses suggest that the causal relationship may be reversed such that the state of obesity alters circulating 25(OH)D concentrations. These mechanisms may include volumetric dilution over greater mass (31), greater storage of 25(OH)D in adipose tissue, which reduces circulating levels (32), or decreased hepatic 25-hydroxylase activity that reduces bioactivity by suppressing 25-hydroxylation (33). 25(OH)D receptor polymorphisms have been associated with increased cholesterol and triglycerides, with the proposed pathway involving regulation of the synthesis of bile acid (34); 25(OH)D effects on lipid metabolism may also occur via its role in regulating calcium and parathyroid hormone (34). Furthermore, evidence suggests that 25(OH)D contributes to the regulation of pancreatic β-cell function through the expression of a calcium-binding protein. The latter protects against cytokine-mediated cell death, which is consistent with in vivo findings that link vitamin D deficiency to impaired insulin secretion and glucose tolerance (35).

### Strengths and limitations

A strength of our project is that it was conducted in a prospectively followed cohort of American Indian populations in 3 regions of the US, with assessment of risk factor incidence at 6 and 13 years of follow-up. However, the cohort contained relatively few adolescents, so power to assess associations was limited; larger studies with young American Indians are needed to assess the potential relationship. In addition, data for the 13-year follow-up were not available for incident obesity, any dyslipidemia, and metabolic syndrome. Diabetes at second follow-up was not directly measured by clinical assessment but defined according to medical record confirmation of self-reported diagnoses; thus, potential exists for nondifferential misclassification. In addition, 25(OH)D measurements were conducted on samples that were stored for 11 to 13 years before measurement. However, previous investigators have determined that 25(OH)D is stable under usual storage conditions of −80°C (36,37). Furthermore, if samples were degraded due to long-term storage, we would likely observe fewer participants with vitamin D deficiency and therefore the result would be biased toward the null (36). The larger SHFS and this substudy were designed to fill a gap in the literature on heart disease and its risk factors by including American Indians; thus, the generalizability of study results is limited to American Indian adolescents from SHFS communities. Also, because no clinically relevant definition of vitamin D deficiency has been established for optimal cardiovascular health, we used 25(OH)D ≤ 20 ng/mL, which is recommended by the Institute of Medicine for optimal bone health (15). Some literature suggests that levels should be at least 25 ng/mL to 30 ng/mL for extra skeletal benefit (1,15); however, increasing the serum cut point to 25 ng/mL to 30 ng/mL would have increased the prevalence of vitamin D deficiency, thus making our analysis involving 20 ng/mL more conservative. Finally, the prevalence of vitamin D deficiency was higher in participants from Arizona, which was unexpected because, compared with participants from Oklahoma and the Dakotas, Arizona participants theoretically may receive more sunlight due to the climate in Arizona. However, perhaps people in Arizona spend more time inside due to the high temperatures. More studies are needed with direct measurements of sunlight exposure.

### Conclusions and implications for public heath practice

We demonstrated that vitamin D deficiency in adolescence is related to measures of obesity, low HDL-C, and metabolic syndrome in American Indian populations, which is consistent with other US populations (4,5). Furthermore, vitamin D deficiency early in life may be associated with the development of diabetes later in life; however, larger studies with longer follow-up are needed to confirm this observation. Because of these observations and because vitamin D deficiency is amenable to changes in diet and vitamin supplementation, the results of this study could provide evidence for public health strategies designed to reduce vitamin D deficiency. These could include community health programs targeting vitamin D supplementation among American Indian adolescents. In addition, community education programs on the benefits of vitamin D supplementation, consuming foods high in vitamin D, and getting adequate amounts of sun exposure may reduce the high levels of vitamin D deficiency that we observed among American Indian adolescents.
